# Ongoing measles outbreak, the Federation of Bosnia and Herzegovina, December 2023 to February 2024

**DOI:** 10.2807/1560-7917.ES.2024.29.9.2400107

**Published:** 2024-02-29

**Authors:** Sanjin Musa, Irma Salimović-Bešić, Jasmina Brkić Džambić, Nijaz Tihić, Anisa Bajramović, Suzana Arapčić, Amela Dedeić-Ljubović, Siniša Skočibušić

**Affiliations:** 1 Department of Infectious Disease Epidemiology, Institute for Public Health of the Federation of Bosnia and Herzegovina, Sarajevo, Bosnia and Herzegovina; 2 Sarajevo School of Science and Technology Sarajevo Medical School, Sarajevo, Bosnia and Herzegovina; 3 Unit for Clinical Microbiology, Clinical Centre of the University of Sarajevo, Sarajevo, Bosnia and Herzegovina; 4 Faculty of Health Studies, University of Sarajevo, Sarajevo, Bosnia and Herzegovina; 5 Department of Epidemiology, Institute for Public Health of Tuzla Canton, Tuzla, Bosnia and Herzegovina; 6 Department of Microbiology, Polyclinic for Laboratory Diagnostics, University Clinical Centre Tuzla, Tuzla, Bosnia and Herzegovina; 7 Faculty of Medicine, University of Tuzla, Tuzla, Bosnia and Herzegovina; 8 Department of Epidemiology, Institute for Public Health of Sarajevo Canton, Sarajevo, Bosnia and Herzegovina

**Keywords:** measles outbreak, MMR vaccine, Federation of Bosnia and Herzegovina, Bosnia and Herzegovina

## Abstract

We report on an ongoing measles outbreak in the Federation of Bosnia and Herzegovina with 141 cases notified between week 52 2023 and week 6 2024. Among those with known vaccination status, 97% were unvaccinated and the most affected group is children under the age of 5 years (n = 87) who were not vaccinated during the pandemic years. Sixty-eight cases were hospitalised, the most common complications were measles-related pneumonia and diarrhoea. The sequenced measles viruses from four cases belonged to genotype D8.

The Federation of Bosnia and Herzegovina (FBiH), an entity of Bosnia and Herzegovina (BiH), has faced challenges in attaining the necessary vaccination coverage to eliminate measles in the past, and the coronavirus disease 2019 (COVID-19) pandemic has had a further negative effect on the routine immunisation programme of children and adolescents. We report on the third and still ongoing major measles outbreak in FBiH in the past 10 years which began in the northern region of the country, in Tuzla Canton, in December 2023. As of 12 February 2024, 141 cases of measles have been notified in four of the 10 cantons of FBiH.

## Surveillance of measles and measles-mumps-rubella (MMR) vaccination

Notification of a patient with measles is compulsory by law in FBiH. Case classification is based on the European Union case definitions of measles [1]. Healthcare workers must notify cases of measles to local and cantonal public health authorities for investigation and implementation of control measures. Blood samples and/or nasopharyngeal swabs are taken from possible cases and analysed in cantonal laboratories and the federal laboratory for measles (Unit for Clinical Microbiology, Clinical Centre of the University of Sarajevo).

**Figure 1:**
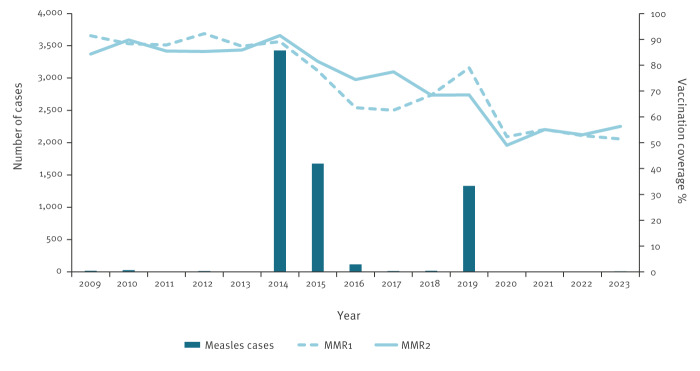
Notified measles cases and coverage of measles-mumps-rubella vaccination in children, the Federation of Bosnia and Herzegovina, 2009–2023 (n = 6,662)

MMR: measles-mumps-rubella; MMR1: children < 24 months of age immunised with one dose of measles-mumps-rubella vaccine; MMR2: children of the age of 6 years immunised with two doses of measles-mumps-rubella vaccine.

At the federal level, the Institute of Public Health of FBiH collects and analyses notifications of measles cases from cantonal institutes and laboratories by using federal surveillance data recording. In FBiH, routine childhood vaccinations are mandatory and free of charge. A combined measles-mumps-rubella vaccine (MMR) is given to children at 12 months and 6 years of age. Figure 1 shows the number of notified measles cases and vaccination coverage for MMR since 2009.

## Epidemiological investigation

In week 52 2023, six hospitalised cases of measles (Cases 1–6) were notified. These cases were laboratory-confirmed at the University Clinical Centre. Four cases (Case 1–4) were born in 2022 and attended the same preschool, Case 5 was born in 2017, and Case 6 in 2012. There was no epidemiological link between the preschool cases and Cases 5 and 6, which were from different other municipalities of the same canton.

An investigation was initiated after the notifications of Cases 1–6. One child attending the preschool developed symptoms early December 2023, but the illness was not recognised as measles by healthcare workers. Of the 38 children who attended the same preschool, 29 were unvaccinated and six were younger than 12 months, thus below the age of the first MMR vaccination. In the days following symptom onset of the index cases, six new measles cases associated with them were notified.

**Figure 2:**
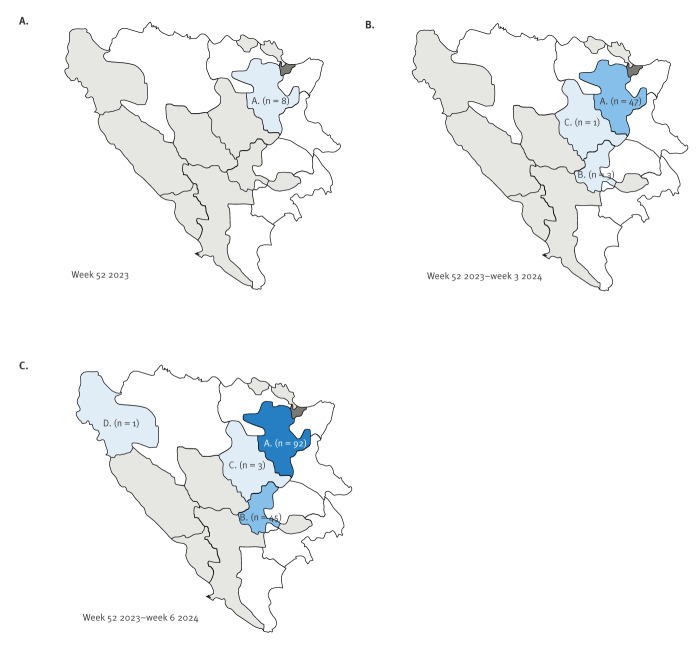
Notification of measles cases by cantons, the Federation of Bosnia and Herzegovina, week 52 2023–week 6 2024 (n = 141)

In week 3 2024, three children from a preschool in another canton were notified. The first of these three cases developed symptoms on 11 January and was initially diagnosed as scarlet fever. One child was notified in a third canton (Figure 2). These cases were epidemiologically linked with cases in the first canton.

The higher intensity of the blue colour represents an increasing number of notified cases (provided in the brackets). The medium grey regions represent cantons of the entity of the Federation of Bosnia and Herzegovina (FBiH) with no notified measles cases. The white areas within FBiH represent the entity of the Republic of Srpska, while the dark grey region belong to the Brčko District (data not included).

**Figure 3:**
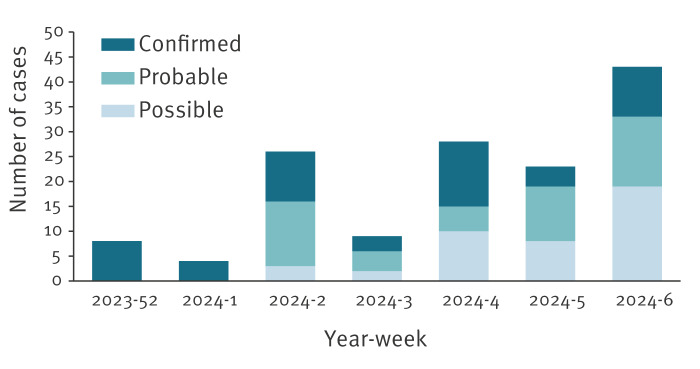
Possible, probable and confirmed measles cases by week, the Federation of Bosnia and Herzegovina, week 52 2023–week 6 2024 (n = 141)

**Figure 4:**
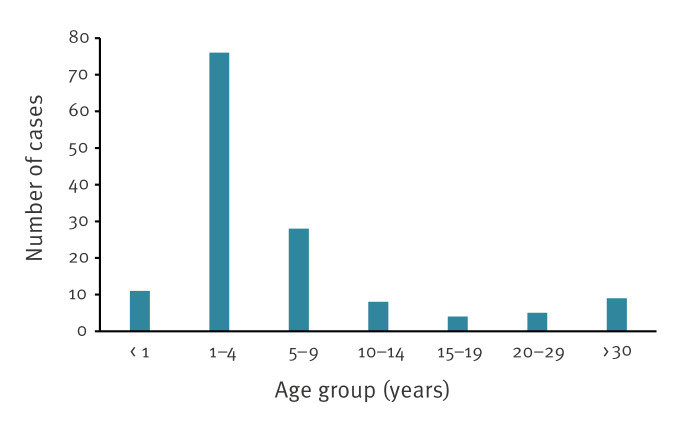
Age distribution of notified measles cases, the Federation of Bosnia and Herzegovina, week 52 2023–week 6 2024 (n = 141)

In total, between week 52 2023 and week 6 2024, 141 measles cases were notified, including 52 (36.9%) laboratory-confirmed cases (Figure 3). Measles cases were notified in four of the ten cantons of FBiH. The index case in one canton had a history of travel to Austria and contact with a measles case there. Overall, the most affected age group was children aged 1–4 years, with 76 cases (53.9%) (Figure 4). Of the 141 notified measles cases, 130 (92.2%) were unvaccinated, four (2.8%) had incomplete vaccination (one dose of MMR) and for seven (5.0%), this information was not available. During this period, 68 (48.2%) cases were hospitalised. Of the hospitalised cases, six (8.8%) were < 1 year, 31 (45.6%) were 1–4 years, 19 (27.9%) were 5–9 years, four (5.9%) were 10–14 years and eight (11.8%) were aged ≥ 20 years. The most common complications were measles-related pneumonia and diarrhoea. No deaths were reported.

## Laboratory investigation

Serum samples from 52 cases tested positive for IgM antibodies against the measles virus at the Department of Microbiology, Polyclinic for Laboratory Diagnostics, University Clinical Centre Tuzla (Anti-Measles Virus Abs, Orgentec Diagnostika, Mainz, Germany) and the Unit for Clinical Microbiology, Clinical Centre of the University of Sarajevo (SERION ELISA classic Masern/Measles Virus IgM, SERION Diagnostics, Wurzburg, Germany). For confirmation, we analysed at the Unit for Clinical Microbiology, Clinical Centre of the University of Sarajevo, the World Health Organization (WHO) accredited laboratory for measles and rubella, nasopharyngeal swab specimens taken from 15 cases serologically positive for measles virus. Measles virus RNA was confirmed by real-time reverse transcription (RT)-PCR (The Centers for Disease Control and Prevention (CDC), Atlanta, United States (US) Measles Virus Detection kit). Genotype determination was done from four measles-RNA-positive samples by sequence analysis of the 450 nucleotide (nt) fragment coding the C-terminal of the nucleoprotein (N) (CDC Measles Virus Genotyping kit) according to the WHO manual [2]. All sequences were submitted to the GenBank (https://www.ncbi.nlm.nih.gov/genbank/) sequence database (accession numbers: PP274936, PP274937, PP274938, and PP274939) and to the World Health Organization Global Measles and Rubella Laboratory Network (MeaNS2) Measles Virus Nt Surveillance database (https://who-gmrln.org/means2) (case ids: 153953, 153954, 153955, 153956).

**Figure 5:**
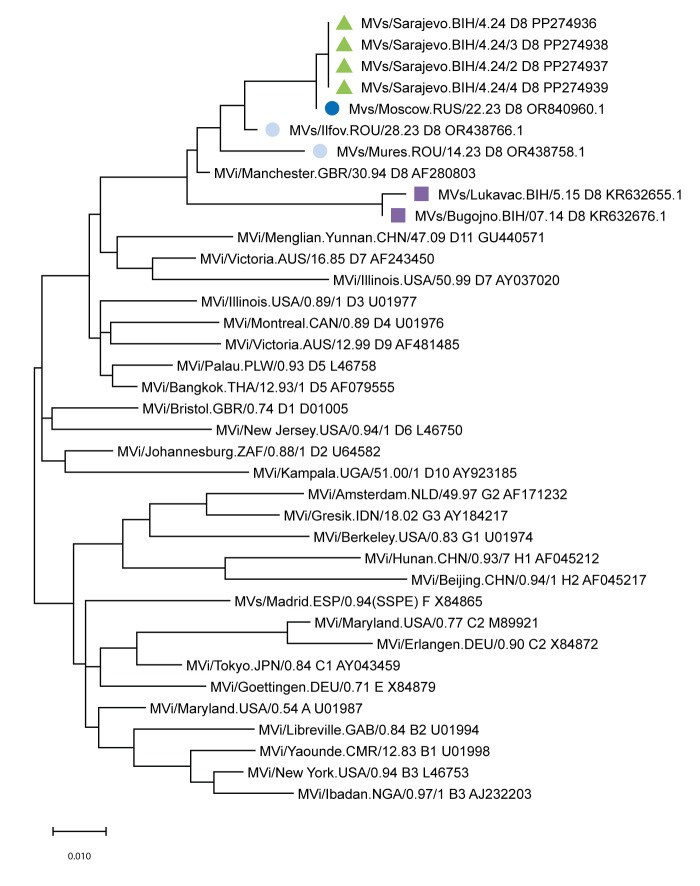
Phylogenetic analysis of measles viruses involved in a measles outbreak, the Federation of Bosnia and Herzegovina, week 52 2023–week 6 2024

All four sequences were identical and belonged to the D8 genotype of the measles virus. The most similar sequence to these was the one from Moscow, Russia, reported in week 28 2023 with a difference of one nt (GenBank, OR840960.1). The currently circulating D8 variant in FBiH differs from those isolated in Romania in 2023 [3] and in BiH during the two-wave outbreak in 2014–2015 [4] (Figure 5).

WHO: World Health Organization.

The evolutionary history was inferred by using the Maximum Likelihood method and Tamura-Nei model by applying Neighbour-Join and BioNJ algorithms to a matrix of pairwise distances estimated using the Tamura-Nei model [18,19], and then selecting the topology with superior log likelihood value. Evolutionary analyses were conducted in MEGA X using 450 nucleotide coding for the C-terminal 150 amino acids of the measles virus nucleoprotein. The phylogenetic tree includes sequences from this outbreak (green triangles), WHO reference sequences (non-marked), the Basic Local Alignment Search Tool (BLAST+; version 2.15.0) fits (blue circles) and two variants of the D8 virus genotype from an outbreak in 2014–2015, the Federation of Bosnia and Herzegovina [4] (purple squares).

## Discussion

With 30,000 measles cases notified in 40 of its 53 member countries, the WHO has noted an alarming increase in measles cases in the WHO European Region in 2023. One major reason for this recurrence is the decreased vaccination rates during the pandemic years 2020–2022 [5]. In 2023, an outbreak, still ongoing, was detected in Romania with new D8 virus variants identified [3]. In January 2024, outbreaks of measles were reported in the United Kingdom [6].

In the 2014‒2015 measles outbreak in FBiH, 5,103 cases were notified, with preschool and school children and adolescents of 5–19 years as the most affected age groups [7]. All sequences belonged to genotype D8 in the 2014–2015 outbreak [4]. In the 2019 outbreak in FBiH, 1,332 cases were notified, most among 0–5-year-olds and all sequences from the analysed case samples belonged to genotype B3 [8].

Routine childhood vaccination uptake in FBiH has been steadily declining since 2014. In 2019, the reported coverage was improved. The coverage of the third dose of the diphtheria-tetanus-pertussis containing vaccine (DTP3) was 80.2% in < 1-year-olds and of the first dose of MMR (MMR1) it was 79.0% in < 2-year-olds [8], and below the 95% target required for measles and rubella elimination in European Immunization Agenda [9].

Even though childhood immunisation is mandatory in FBiH, it is clearly not being implemented, meaning healthcare workers and primary healthcare centres do not routinely report refusals. According to the FBiH vaccination policy document, verification of the status of vaccinations at the time of enrolment in all preschool programmes is required, at all school levels (from elementary to high school) and during every medical appointment. In accordance with the law, parents who choose not to vaccinate their children will face financial fines.

With the development of the COVID-19 pandemic and its subsequent repercussions, in 2020, the coverage of DTP3 decreased by 17.6% and MMR1 by 26.7% [10,11]. Similar coverages were registered in 2021 and 2022 [10]. Estimates of coverage do not include children who have delayed vaccination (in 2022, for instance, delayed MMR1 vaccination received 15% of children). There were six measles cases notified between 2020 and 2022 [10].

Globally, the estimated coverage in 2020 fell to 76.7% for DTP3 and measles-containing vaccine (MCV1) dropped to 78.9% [12]. Between 2021 and 2022, the global MCV1 coverage increased from 81% to 83%, yet remained below the 2019 coverage level (86%), with higher achieved MCV1 coverage of 93% in the WHO European Region [13].

To address the underlying causes of suboptimal vaccination, the Institute for Public Health of FBiH implemented a WHO Tailoring Immunization Programmes (TIP) project between 2017 and 2019 [14]. Local data on barriers and drivers to childhood vaccination behaviours are available to inform strategies, interventions and communications [15-17]. Further efforts are needed to ensure adequate routine immunisation coverage.

In response to the current outbreak, specific control measures include strengthening surveillance, communication with the public and healthcare professionals and catch-up vaccination activities. There has been an increase in demand for MMR vaccinations since the beginning of the outbreak (the number of MMR vaccinations increased by 67% or 1,699 doses in January 2024 compared with January 2023). Two cantons declared measles outbreak. To improve accessibility to vaccination services, healthcare authorities in the affected cantons urged primary care centres to provide drop-in vaccination sessions, extended opening hours and allocated whole days for vaccination of school and preschool children. With registered low vaccine uptake in previous years, there is a high risk of spreading the current measles outbreak to other municipalities and cantons of FBiH.
